# Impact of Rehabilitation Intensity on 3-Year Mortality among Children with Moderate to Severe Cerebral Palsy: A Population-Based Cohort Study

**DOI:** 10.3390/ijerph18189932

**Published:** 2021-09-21

**Authors:** Chiao-Lin Hsu, Chia-Ling Hung, Shih-Ju Huang, Chun-Hao Yin, Chi-Hsiang Chu, Tsu-Jen Kuo, Yao-Min Hung

**Affiliations:** 1 Center of Health Management, Kaohsiung Veterans General Hospital, Kaohsiung 81362, Taiwan; jolindr0610@gmail.com; 2 Department of Family Medicine, Kaohsiung Veterans General Hospital, Kaohsiung 81362, Taiwan; 3 Center for Geriatrics and Gerontology, Kaohsiung Veterans General Hospital, Kaohsiung 81362, Taiwan; 4 Department of Physical Medicine and Rehabilitation, E-Da Cancer Hospital, Kaohsiung 82445, Taiwan; clhung1989@gmail.com; 5 Department of Pediatrics, Kaohsiung Veterans General Hospital, Kaohsiung 81362, Taiwan; stewisme@vghks.gov.tw; 6 Department of Medical Education and Research, Kaohsiung Veterans General Hospital, Kaohsiung 81362, Taiwan; mo521141@gmail.com; 7 Institute of Health Care Management, National Sun Yat-sen University, Kaohsiung 80424, Taiwan; 8 Department of Statistics, Tunghai University, Taichung 407224, Taiwan; loveweib@gmail.com; 9 Department of Marine Biotechnology and Resources, National Sun Yat-Sen University, Kaohsiung 80424, Taiwan; 10 School of Dentistry, Chung Shan Medical University, Taichung 40201, Taiwan; 11 Department of Dentistry, Chung Shan Medical University Hospital, Taichung 40201, Taiwan; 12 Department of Internal Medicine, Kaohsiung Municipal United Hospital, Kaohsiung 80457, Taiwan; 13 Institute of Medicine, Chung Shan Medical University, Taichung 40201, Taiwan; 14 College of Health and Nursing, Meiho University, Pingtung 912, Taiwan

**Keywords:** cerebral palsy, rehabilitation, mortality, nationwide cohort study

## Abstract

Though numerous studies demonstrated the positive effect of rehabilitation on cerebral palsy (CP) children, there was no literature addressing the role of rehabilitation on mortality among children with CP. Therefore, we aimed to evaluate the impact of rehabilitation intensity on mortality among children with moderate to severe CP. This retrospective cohort study was conducted by National Health Insurance Research Database in Taiwan. Children (<12 years) with newly diagnosed moderate to severe CP between 1 January 2000 and 31 December 2013 were included. All patients were followed up for 3 years after CP diagnosis or death or until 31 December 2013. The intensity of rehabilitation therapy within 6 months after CP diagnosis was categorized into <6 times and ≥6 times. The Cox proportional hazard analysis was used to determine the association between rehabilitation intensity and all-cause mortality after adjusting age, sex, other demographic factors and comorbidities. Among 3936 severe CP children, 164 (4.2%) died during the 3-year follow-up period. The mortality rate was higher among patients receiving rehabilitation < 6 times within 6 months than those ≥6 times within 6 months after adjusting demographic profile and comorbidities (adjust HR (aHR): 1.96, 95% CI 1.33–2.89, *p* < 0.001). We found that patients who were younger (aHR: 0.84, 95% CI 0.76–0.92, *p* < 0.001), who were receiving inpatient care more than twice in 1 year before their CP diagnosis (aHR: 2.88; 95% CI: 1.96–4.23; *p* < 0.001), and who have pneumonia (aHR: 1.41, 95% CI 1.00–1.96, *p* = 0.047), epilepsy (aHR: 1.41, 95% CI: 1.02–1.95, *p* = 0.039) and dysphagia (aHR: 1.55, 95% CI: 1.06–2.26, *p* = 0.024) have higher risk of mortality. Rehabilitation ≥ 6 times within 6 months has a potentially positive impact on pediatric CP survival. Besides having a younger age, being hospitalized more than twice within a year before diagnosis and having pneumonia, epilepsy and dysphagia were modifiable risk factors in clinical practice for these children.

## 1. Introduction

Cerebral palsy (CP) has been recognized as damage to the immature brain that leads to poor motor function coordination, aberrant movement and posture development, and impaired perception. These worsen a child’s health outcome, quality of life and lifespan [1]. A recent study showed that nearly half of CP patients died by 15 years old among severely disabled CP [2]. The mortality of children with CP rises gradually in the first five years of life [3]. Moreover, the lifespan among children with CP remains lower than the general population [4].

Health or social caregivers and families who have CP children often pay special attention to CP-associated diseases, which can lead to hospitalization and be life-threatening. Most of the deaths among children with CP were attributed to modifiable risk factors, including respiratory problems [5], epilepsy [6], oropharyngeal dysphagia [7], feeding problems [8] and cognitive impairments [9]. This raises the question of whether some of the deaths could have been avoided by interventions such as rehabilitation to improve swallowing, prevent aspiration and strengthen body muscle.

According to the recent CP management guideline [10], referring children for integrated rehabilitation is an urgent task for pediatricians when CP is diagnosed. Early rehabilitation has aimed to optimize motor [11], cognition, communication and social-psychological outcomes by capitalizing on neuroplasticity [12]. The central nervous system was reported to have potential plasticity and reorganization throughout life, and rehabilitation efforts were reported to enhance all domains of function and maximize long-term independence [13]. Thus, in addition to conventional neurodevelopmental and motor learning-based strategies focusing on body structures impairment, the current multidisciplinary rehabilitation approach (including physical, occupational, cognition, psycho-social and swallowing therapy) [14] is a superior and essential way to achieve daily activity independence, enhance social participation and improve quality of life.

Research has shown the positive effect of rehabilitation on mortality for conditions such as chronic obstructive pulmonary disease [15], cardiovascular disease [16] and osteoporotic fracture [17]. Nevertheless, there was no literature addressing the role of rehabilitation on mortality among children with CP.

Therefore, the present study aims to fill a gap in the literature by investigating the association between rehabilitation and mortality in children with severe CP. To this end, we assessed the impact of rehabilitation and modifiable factors associated with 3-year mortality among children with severe CP.

## 2. Methods

### 2.1. Data Source

Data for this retrospective cohort study were obtained from the National Health Insurance Research Database (NHIRD) from 1 January 2000 to 31 December 2013. Taiwan’s National Health Insurance (NHI) was established in 1995 and covered up to 99.99% of Taiwan’s population. The NHIRD was released for research purposes and provided thorough population-based evidence to support clinical decisions [18,19]. The NHIRD comprises comprehensive demographic characteristics, clinic visits and hospitalization dates, disease diagnosis codes, procedure codes, prescription codes and medical costs for reimbursement. The Registry of Catastrophic Illnesses Patient Database (RCIPD) is one part of NHIRD [20] encompassing some 30 diseases, including CP.

This study was approved by the Institutional Review Board of Kaohsiung Veterans General Hospital, Kaohsiung in Taiwan (VGHKS15-CT12-01). Informed consent was not obtained due to all personal identifications being replaced with surrogate numbers.

### 2.2. Study Population

All patients younger than 12 years old with a new diagnosis of severe CP (identified by catastrophic illness) between 1 January 2000 and 31 December 2013 were enrolled from the NHI dataset and RCIPD. The index date was defined as the date of CP diagnosis since once CP is diagnosed, pediatricians arrange rehabilitation as soon as possible according to the public health policy. We excluded patients who died too soon (within 6 months rehabilitation period). Taiwan’s rehabilitation system typically allows patients to undergo it a maximum of three times a day; therefore, more than 540 rehabilitation times within six months would be deemed inconsistent with current medical conditions in Taiwan. It is not clear if there may be errors in the data input due to administrative or system problems, but these data were considered to be inaccurate, and they were deleted.

### 2.3. Moderate to Severe CP Diagnosis

Moderate to severe CP diagnoses in this study were based on the International Classification of Disease, Ninth Revision, Clinical Modification (ICD-9-CM) diagnosis code 343.X and an NHI-issued catastrophic illness certificate that was validated in previous research articles [21]. Catastrophic illness certificates were administered by National Health Insurance Administration. CP children with moderate to severe physical or mental disability and verified by pediatric neurologists or rehabilitation specialists can apply for a catastrophic illness certificate. The disability severity was based on the International Classification of Functioning, Disability and Health, which includes: 1. Cognition, coherence and psychological level; 2. Joint mobility; 3. Muscle strength loss; 4. Gross Motor Function Classification grades.

Patients who had their CP catastrophic illness certificate approved when they were over age 12, whose data were incomplete, or who died within six months of their CP diagnosis were excluded.

### 2.4. Variables

Only the patients who survived over 6 months after a CP diagnosis were included in the study. Those who received rehabilitation ≥6 times within 6 months were then regarded as the high-intensity rehabilitation cohort, and patients who never received rehabilitation or received <6 times within 6 months were assigned to the low-intensity rehabilitation cohort. We chose 6-months-rehabilitation on the basis of current clinical guidelines for pediatric care [22] and rehabilitation intensity cut-off values discriminatory ability (Table S1).

The amount of physical, occupational and speech therapy services after CP diagnosis was retrieved from outpatient clinic visits. Each rehabilitation time is 30 min.

Patients’ sociodemographic characteristics, including CP diagnosis age (based on RCIPD), gender, hospital level (medical center, regional hospital and others), residential region (North, Middle and others) and urbanization (rural and others), were obtained from enrolment data.

Several coexisting morbidities that may associate with mortality were evaluated when we followed up for outcome. Inpatient care, more than two times within 1 year before CP diagnosis, was assessed and retrieved from inpatient claims and ambulatory data. Other comorbidities in the analysis were epilepsy (ICD-9-CM codes 345), congenital heart disease (ICD-9-CMcode 746.9), scoliosis (ICD-9-CMcodes 737.43, 737.3, 737.34, 737.30, 737.32, 754.2), GERD (ICD-9CM codes 530.85, 530.11, 530.81), dysphagia with tube feeding (ICD-9CM codes 438.82, 787.2, 280.8 and NHI treatment code 54032C), perinatal complications including preterm labor and small size for gestational age (ICD-9CM codes 760–764, 765–765.19, 766–779, V137), traumatic brain injury (ICD-9CM codes 800–804, 850–854) and intellectual disability (ICD-9CM codes 317, 318, 319). The ICD-CM codes occurring in the inpatient setting or in ≥3 ambulatory care claims for a chronic disease or ≥1 ambulatory care claims for the acute disease were enrolled.

### 2.5. Outcome

The main measured outcome is all-cause mortality, which is defined by disenrollment from the NHI due to death. The follow-up time was defined as the period from the index date to the death, or withdrawal from the NHI program, or 3 years, whichever came first.

### 2.6. Statistical Analysis

The chi-squared test was used for categorical variables and the t-test for continuous variables.

Univariate Cox proportional hazards models were applied to depict survival over time by each of the independent variables. Then we used the multivariable Cox proportional hazards models to assess independent variables associated with mortality. All recorded variables were considered in estimating hazard ratios (HRs) with a 95% confidence interval (CI). All variables were entered in a multivariate Cox proportional hazards regression model by the step-forward method. A *p*-value less than 0.05 was considered statistically significant.

The Kaplan–Meier method was used to estimate the cumulative survival between the groups during the follow-up period. The log-rank test was performed to compare the outcome distributions of two cohorts.

All statistical analysis was conducted using Statistical Analysis Software (SAS) version 9.4 (SAS System for Windows, SAS Institute, Cary, NC, US) and SPSS (version 20; SPSS Inc., Chicago, IL, USA).

## 3. Results

**Figure 1:**
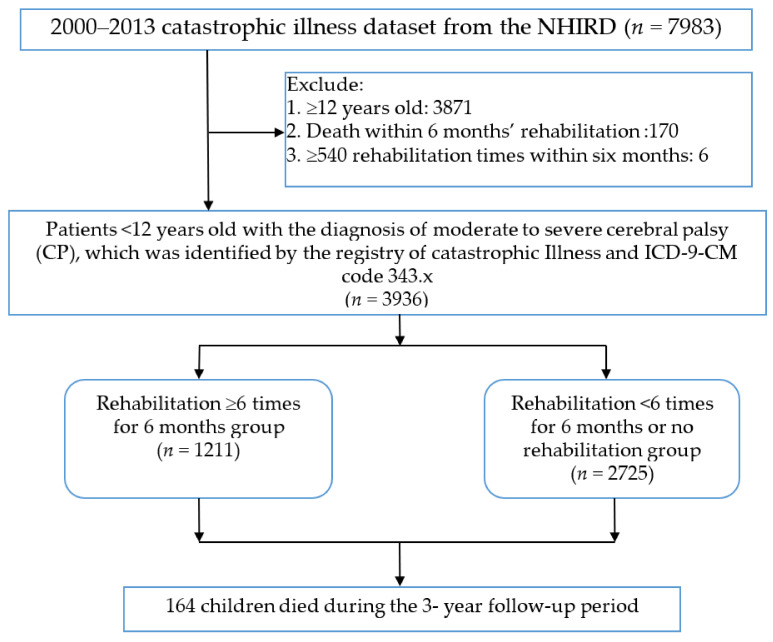
Population flowchart. Patients with moderate to severe cerebral palsy diagnoses were enrolled from the NHIRD dataset. Patients were divided into two groups according to rehabilitation frequency. Abbreviation: NHIRD: National Health Insurance Research Database; CP: cerebral palsy.

The study population included 3936 newly diagnosed moderate to severe CP children. In total, 1211 (30.8%) CP children received rehabilitation ≥6 times within 6 months, and 2725 (69.2%) severe CP children did not receive rehabilitation or received rehabilitation <6 times within 6 months. (Figure 1) Of those, 464 (11.8%) died during the whole period (median, 3.8 years, until 31V December 2013). A total of 164 (4.2%) children died during the 3-year follow-up period. Among the 164 deceased CP children, 127 (5%) were in the low rehabilitation group, and 37 (2.6%) were in the high rehabilitation group, *p* < 0.001. The mean age in Table 1 indicates the age of obtaining catastrophic illness certification, which is the age of CP diagnosis. The mean age of the high-intensity rehabilitation cohort was 3.17 ± 2.2) years and 3.68 ± 2.9 for the low-intensity cohort. Age (*p* < 0.001), hospital character (*p* < 0.001), residential region (*p* < 0.001), and comorbidities including pneumonia (*p* = 0.001), epilepsy (*p* < 0.001), perinatal complication (*p* < 0.001), GERD (*p* = 0.001), dysphagia (*p* = 0.006) and intellectual disability (*p* < 0.001) showed a significant difference between the high intensity rehabilitation cohort and the low intensity rehabilitation cohort (Table 1).

Table 2 shows the crude and adjusted hazard ratios by Cox proportional hazard analysis. In univariate analysis, the following factors were statistically significantly associated with mortality, younger age (HR: 0.71; 95% CI 0.64–0.79; *p* < 0.001) and low rehabilitation intensity (HR: 1.55; 95% CI 1.24–1.95; *p* < 0.001), requiring inpatient care more than two times within 1 year before a severe CP diagnosis (HR: 5.46; 95% CI 3.94–7.56; *p* < 0.001, with comorbidities of pneumonia (HR: 2.34; 95% CI 1.72–3.19; *p* < 0.001), epilepsy (HR: 2.14; 95% CI: 1.56–2.94; *p* < 0.001), congenital heart disease (HR: 2.06; 95% CI: 1.01–4.18; *p* = 0.047), dysphagia (HR: 3.21; 95% CI: 2.26–4.56; *p* < 0.001) and intellectual disability (HR: 0.58; 95% CI 0.34–0.99; *p* = 0.044). After adjustment for covariates by stepwise Cox regression analysis, we found that patients who received low intensity rehabilitation within 6 months (HR: 1.96; 95% CI 1.33–2.89; *p* < 0.001) had a higher risk of mortality. Other factors that had higher mortality risk include younger age (HR: 0.84; 95% CI 0.76–0.92; *p* < 0.001), receiving inpatient care more than twice within one year before a severe CP diagnosis (HR: 2.88; 95% CI: 1.96–4.23; *p* < 0.001), and comorbidities of pneumonia (HR: 1.41; 95% CI 1.00–1.96; *p* = 0.047), epilepsy (HR: 1.41; 95% CI 1.02–1.95; *p* = 0.039) and dysphagia (HR: 1.55; 95% CI 1.06–2.26; *p* = 0.024) (Table 2).

**Figure 2:**
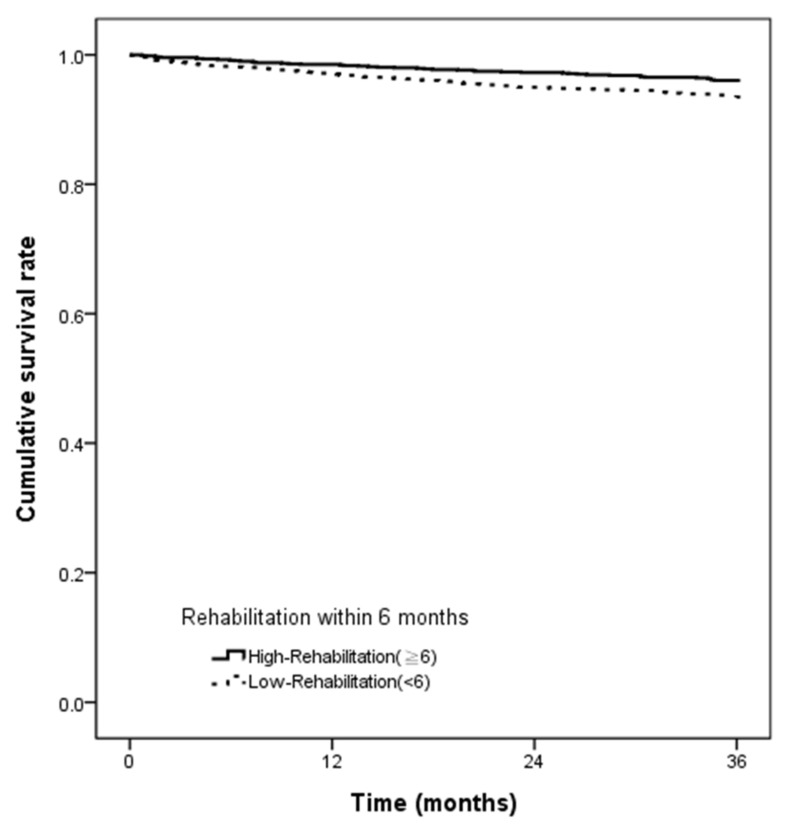
Demonstrated the Kaplan–Meier estimates of survival rate stratified by rehabilitation intensity within 6 months.

Figure 2 demonstrates the Kaplan–Meier analysis. The survival rate was significantly different between the two groups (high rehabilitation groups vs. low rehabilitation groups) since the index date. (The log-rank test of *p* < 0.001 (Figure 2).)

## 4. Discussion

To the best of our knowledge, this is the first cohort study using nationwide population-based data to assess the impact of rehabilitation intensity on pediatric CP mortality. Our study comprised strict inclusion criteria and longer follow-up periods. We found that ≥6 times rehabilitation within 6 months (approximately monthly frequency rehabilitation) have a positive association with mortality. Younger age, having been previously hospitalized more than twice within one year before a severe CP diagnosis, and with a history of pneumonia, epilepsy or dysphagia were significant predictors for mortality among children with severe CP.

Though the rehabilitation intensity in our study was lower than the recent guideline suggested (physical frequency one to two times per week for six months) [22], the present study provides additional evidence that rehabilitation intensity has a positive association with mortality. We implied that rehabilitation improves ambulation (a strong predictor of survival among CP) [4], and swallowing ability [23] contributed to the positive association.

Furthermore, one randomized controlled trial conducted by Bower et al. [24] suggested a potential non-essential role of higher intensity of therapy up to 1–2 times per week. They assumed that the loading and demanding of children and their families would undermine the efficacy of intensive therapy.

**Figure 3:**
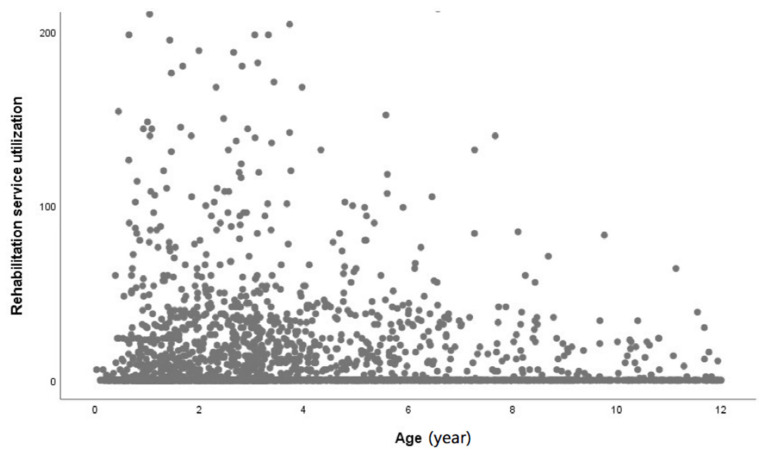
The presented rehabilitation utilization service among moderate to severe CP children. There is a trend that younger severe CP patients have higher rehabilitation service utilization than older individuals.

There is a trend that younger moderate to severe CP patients have higher rehabilitation service utilization than older individuals in Figure 3. Additionally, children with severe CP have a higher rehabilitation rate before the age of six in our observation. This is consistent with another nationwide database study [25] and clinical experience that the younger a person is when CP is diagnosed, the more severe the CP is, and there will be more complications requiring intervention. In Taiwan, the Protection of Children and Youths Welfare and Rights Act requires local governments to offer early rehabilitation services to CP patients under six years old. This medically oriented program offers integrated evaluation, treatment and rehabilitation services for children with a diagnosis of intellectual disabilities or developmental delay. However, only 30.8% of the moderate to severe CP patients received rehabilitation, which does not match Taiwan’s health policy. We presume this is because (1) the patient’s physical condition was too weak to engage in rehabilitation or no potential to recover (such as vegetative or bed-ridden status) (2); the patient’s mental condition was too poor to expect benefits from rehabilitation (3); the accessibility of rehabilitation resources may be limited in types of hospitals where the patient was from a family whose low income prevented them from receiving medical services; (4) parents were not aware of any signs and delayed seeking medical attention, or did not follow the advice from a pediatrician (5); different case payment in different hospital level may limit physician’s referral rate. This finding deserves clinicians’ and the government’s awareness. Furthermore, health professionals are encouraged to be proactive in referral.

As in previous studies, younger age [26] carries a higher risk of mortality. CP patients who were admitted to the hospital more than twice within one year before a severe CP diagnosis showed a higher risk of mortality. Blackmore et al. demonstrated that patients who are admitted to the hospital more than once for respiratory reasons are more likely to require hospitalization in the future [27]. Chung and Kenyon et al. also indicated that the previous admission was a risk factor for pediatric readmission [28]. These observations suggest that greater disease severity and prior admission associated with plays an essential role in mortality [28].

We found that CP children have pneumonia history have a higher risk of mortality. Though global child mortality is reducing, children die because pneumonia remains high [29]. As well as CP children, pneumonia and associated respiratory failure were also reported as a leading cause of death among CP children [2,30]. Our finding was consistent with previous studies.

Dysphagia is common in CP patients and exposes children to health problems such as food aspiration, malnutrition and respiratory infections [31]. We found dysphagia with tube feeding had a higher risk of mortality. This is the opposite of the result obtained from previous research [32]. Possible explanations were: (1) the need for gastrostomy is more common among children who have poorer health status and respiratory problems than those without [33]. (2) Gastrostomy was reported to delay adverse outcomes such as pneumonia but does not prevent progressive lung injury [34].

As in previous research, epilepsy was associated with reduced life expectancy for CP patients [35]. CP patients with epilepsy were prone to other disabilities such as cognitive impairment and spastic quadriplegia [36], both of which worsened the survival outcome. Additionally, epilepsy is associated with a higher mortality rate in people with severe CP, as reported by Trabacca et al. [14].

Some limitations should be addressed. First, we did not present Gross Motor Function Classification System level because the information was not present in NHIRD. Therefore, ambulation status cannot be measured. However, we used severe illness certificated criteria (including ambulation ability) to define. Second, we did not assess detailed rehabilitation times per day due to the feasibility of green cloud electronic medical records services since 2016. Third, we cannot obtain confounding factors that may affect the outcome, such as birth bodyweight, parental educational level, breastfeeding duration and household environment factors such as cigarette smoking.

From a public health viewpoint, policymakers are encouraged to enforce prevention strategies focusing on this high mortality risk group. Additionally, applying integrated medical care and rehabilitation therapy is warranted.

## 5. Conclusions

There is a lower rehabilitation services usage rate among CP children in Taiwan. For children with severe CP, high-intensity rehabilitation within 6 months after CP diagnosis was associated with a lower 3-year all-cause mortality risk than low-intensity therapy. Those who were young, have a history of inpatient care twice within one year and have pneumonia, epilepsy and dysphagia need to be more concerned population. Therefore, efforts to promote applying high-intensity rehabilitation therapy for these patients to improve the outcome are warranted.

## Figures and Tables

**Table 1:** Baseline characteristics of severe CP children, *n* = 3936.

Variable	Total	Rehabilitation ≥ 6 Times within 6 Months	Rehabilitation < 6 Times within 6 Months	*p*-Value
	*n* = 3936	*n* = 1211 (30.8%)	*n* = 2725 (69.2%)	
Age, years (Mean ± SD)	3.52 ± 2.7	3.17 ± 2.2	3.68 ± 2.9	<0.001
Gender-Male	2342 (60%)	746 (62%)	1596 (59%)	0.074
Mortality rate (3-year follow-up)	164 (4.2%)	127 (5%)	37 (2.6%)	<0.001
Hospital characteristics				
Medical center	2424 (62%)	796 (66%)	1628 (60%)	
Regional	1281 (32%)	276 (23%)	1005 (37%)	
Others	231 (6%)	139 (11%)	92 (3%)	
Region				<0.001
North	2055 (52%)	578 (48%)	1477 (54%)	
Middle	851 (22%)	298 (24%)	553 (20%)	
South/Others	1030 (26%)	335 (28%)	695 (26%)	
Urbanization				0.203
Urban	2252 (57%)	680 (56%)	1572 (57%)	
Suburban	1392 (35%)	450 (37%)	942 (35%)	
Rural	292 (8%)	81 (7%)	211 (8%)	
Comorbidity				
Inpatient care before diagnosis (≥2 times within 1 year)	1095 (28%)	343 (28%)	752 (28%)	0.638
Pneumonia	1437 (37%)	489 (40%)	948 (35%)	0.001
Epilepsy	1679 (43%)	580 (48%)	1099 (40%)	<0.001
Congenital heart disease	96 (2%)	37 (2%)	59 (2%)	0.581
Perinatal complications	2160 (55%)	739 (61%)	1421 (51%)	<0.001
Scoliosis	89 (2%)	35 (3%)	54 (2%)	0.077
GERD	206 (5%)	89 (7%)	117 (4%)	0.001
Dysphagia	1880 (48%)	618 (51%)	1262 (46%)	0.006
Intellectual disability	569 (15%)	221 (18%)	348 (13%)	<0.001

Abbreviation: GERD, Gastroesophageal reflux disease.

**Table 2:** Cox regression analysis for moderate to severe CP children with 3-year survival, *n* = 3936.

Variables	Univariate		Multivariate	
	HR (95% CI)	*p*-Value	aHR (95% CI)	*p*-Value
Age	0.71 (0.64–0.79)	<0.001	0.84 (0.76–0.92)	<0.001
Gender-Male	1.12 (0.82–1.53)	0.472		
Rehabilitation times				
Low (<6) vs. high (≥6)	1.55 (1.24–1.95)	<0.001	1.96 (1.33–2.89)	<0.001
Hospital characteristics Medical center				
Regional	0.73 (0.52–1.04)	0.079		
Others	0.44 (0.18–1.07)	0.070		
Region North				
Middle	0.69 (0.46–1.06)	0.089		
South and Others	0.84 (0.58–1.21)	0.345		
Residential (Rural vs. others)	1.16 (0.67–2.00)	0.604		
Inpatient care before diagnosis (≥2 times within 1 year)	5.46 (3.94–7.56)	<0.001	2.88 (1.96–4.23)	<0.001
Pneumonia	2.34 (1.72–3.19)	<0.001	1.41 (1.00–1.96)	0.047
Epilepsy	2.14 (1.56–2.94)	<0.001	1.41 (1.02–1.95)	0.039
Congenital heart disease	2.06 (1.01–4.18)	0.047		
Perinatal complications	1.37 (0.99–1.88)	0.052		
Scoliosis	0.48 (0.00–4.24)	0.184		
GERD	1.64 (0.93–2.90)	0.085		
Dysphagia	3.21 (2.26–4.56)	<0.001	1.55 (1.06–2.26)	0.024
Intellectual disability	0.58 (0.34–0.99)	0.044		

Abbreviation: HR = hazard ratio; CI = confidence interval; aHR = adjusted hazard ratio; GERD = gastroenterology.

## Data Availability

The application for the dataset may be mailed to the NHRI at nhird@nhri.org.tw or call at +886-037-246166 ext. 33603 for immediate assistance. Office hours: Monday–Friday, 8:00–17:30 (UTC+8).

## Supplementary Materials

The following are available online at https://www.mdpi.com/article/10.3390/ijerph18189932/s1, Table S1: Discriminatory ability among different rehabilitation intensity cut-off values.

## Abbreviations

NHIRD	National Health Insurance Research Database
RCIPD	Registry for Catastrophic Illnesses Patient Database
HR	Hazard ratio
aHR	adjusted hazard ratio
ICD-9-CM	International Classification of Disease, Ninth Revision, Clinical Modification
GERD	Gastroesophageal reflux disease
