# Supplementary material for: Impact of Rehabilitation Intensity on 3-Year Mortality among Children with Moderate to Severe Cerebral Palsy: A Population-Based Cohort Study
Source: Int J Environ Res Public Health. 2021 Sep 21;18(18):9932. doi: 10.3390/ijerph18189932 (PMC8469265; doi:10.3390/ijerph18189932)
Supplement: Supplementary file 1 [file ijerph-18-09932-s001.zip › ijerph-1353476-supplementary.pdf]

Supplementary Materials

Table S1. Discriminatory ability among different rehabilitation intensity cut-off values.

Cut-off Value	AUROC	p-value
4 times within 6 months	0.563	0.007
6 times within 6 months	0.568	0.003
8 times within 6 months	0.556	0.015
